# Downregulated m⁵C regulator NSUN6 enhances proliferation, migration and affects immune regulation in ovarian cancer

**DOI:** 10.1038/s41598-026-53479-3

**Published:** 2026-05-29

**Authors:** Kuan Hu, Wenjing Shi, Zongjiang Zhou, Xinyue Deng, Juanni Li

**Affiliations:** 1 Department of Hepatobiliary Surgery, Xiangya Hospital, Central South University, Changsha, 410008 Hunan China; 2 Department of Pathology, Xiangya Hospital, Central South University, Changsha, 410008 Hunan China; 3 National Clinical Research Center for Geriatric Disorders, Xiangya Hospital, Central South University, Changsha, 410008 Hunan China

**Keywords:** NSUN6, M⁵C, Metastasis, P-AKT, OC, Cancer, Computational biology and bioinformatics, Immunology, Oncology

## Abstract

Ovarian cancer (OC) remains one of the most lethal gynecological malignancies, largely due to late diagnosis and high metastatic potential. RNA 5-methylcytosine (m⁵C) modification has emerged as a key regulatory mechanism in cancer biology, yet the role of m⁵C-related genes such as NSUN6 in OC remains poorly understood. In this study, we first obtained three OC datasets and one m5C-related dataset from the GEO database and identified differentially expressed genes (co-DEGs) using Venn diagram analysis. Kaplan Meier plotter showed that NSUN6 was the only regulator significantly associated with patient prognosis. Additionly, Western Blot, Online database analysis and immunohistochemical staining evaluation showed that NSUN6 was significantly downregulated in OC and negatively correlated with tumor stage, metastasis and survival. Next, we overexpressed NSUN6 in OC cell lines. CCK8, wound healing and Transwell assays demonstrated that NSUN6 overexpression inhibited OC cell proliferation, migration, and invasion. Rescue experiments confirmed NSUN6-mediated suppression of AKT phosphorylation. In vivo xenograft models confirmed the tumor-suppressive effect of NSUN6, showing reduced tumor burden in both intraperitoneal and subcutaneous models. Bioinformatic analyses revealed enrichment in immune-related pathways and correlations with immune infiltration markers, which were further validated using ovarian cancer clinical samples. Collectively, our results demonstrate that NSUN6 is downregulated in OC and suppresses OC proliferation, migration, and invasion by inhibiting AKT activity, highlighting its potential as a therapeutic target in OC.

**Supplementary Information:**

The online version contains supplementary material available at 10.1038/s41598-026-53479-3.

## Introduction

The incidence rate of ovarian cancer (OC) ranks third among gynecological malignancies, following cervical and endometrial cancers^1^. OC is one of the most common malignant tumors in women and poses a serious threat to women’s health^2,3^. Globally, approximately 324,398 individuals are diagnosed with OC each year, with a five-year survival rate of less than 45%^3,4^. In China, there are about 61,100 new cases and 32,600 deaths due to OC annually^5^. The pathogenesis of OC remains unclear, though it is currently believed to be associated with BRCA gene mutations, pregnancy, medications, hormonal factors, and other variables^6^. OC is often insidious, with no specific clinical symptoms in its early stages and a lack of effective screening markers. As a result, most patients are diagnosed at an advanced stage, contributing to the high mortality rate associated with ovarian cancer^7,8^.

RNA modification refers to various chemical modifications occurring on RNA molecules, which participate in the regulation of numerous pathophysiological processes^9^. RNA methylation is a key epigenetic mechanism that regulates post-transcriptional gene expression and is involved in various physiological and pathological processes^10,11^. Among these modifications, 5-methylcytosine (m⁵C) modification is a widely distributed RNA modification in eukaryotes^12,13^. In recent years, m⁵C modification has been identified on transfer RNA (tRNA), ribosomal RNA (rRNA), and messenger RNA (mRNA)^14^. Regulatory factors involved in m^5^C modification in RNA include m^5^C methyltransferases (NSUN1-7, DNMT1, DNMT2, DNMT3A, and DNMT3B) and demethylases (TET2)^14–16^. Additionally, ALYREF and YBX1 have been further confirmed as nuclear mRNA m5C-binding proteins, which stabilize the structure of their target mRNA or promote its output^17,18^. Accumulating evidence have shown that m^5^C is associated with the progression of various cancers^19,20^. For example, the m^5^C methyltransferase NSUN2 is significantly upregulated in gastric cancer and promotes the proliferation, migration, and invasion of gastric cancer cells^21^. ALYREF is upregulated in bladder cancer and promotes glycolytic metabolism and cell proliferation by recognizing m^5^C in the 3’UTR of PKM2 and stabilizing PKM2^22^. In addition, m^5^C modification also plays an important role in OC. For example, NSUN2 catalyzes KRT13 m⁵C modification^23^, promoting OC migration and invasion. DNMT3A/3B interacts with MicroRNA-29b engage in reciprocal negative feedback, leading to OC progression^24^.

NSUN6 belongs to the Nol1/Nop2/SUN domain (NSUN) protein family^25^. Cytoplasmically localized, NSUN6 selectively methylates specific sites on mRNA and tRNA in a sequence- and structure-dependent manner^26^. Among these, NSUN6 has been implicated in various cancer studies. For example, in triple-negative breast cancer, NSUN6 downregulation dysregulates ECM receptor responses via PI3K/AKT and ERK/MAPK pathways, altering tumor metabolism and cell adhesion^27^. Additionally, in glioblastoma, NSUN6 upregulation increases m^5^C accumulation, enhancing tumor sensitivity to temozolomide (TMZ)^28^. Other studies have shown that NSUN6 expression is reduced in lung cancer cells, and overexpression of NSUN6 inhibits lung cancer cell proliferation, migration, and epithelial-mesenchymal transition (EMT). In addition, the role of NSUN6 in diseases such as pancreatic cancer^29^, osteosarcoma^26^, esophageal squamous cell carcinoma^31^, and colon adenocarcinoma^32^ has been verified, but its role in OC remains unclear.

To date, the role of NSUN6 in OC remains unknown. This study will be an innovative investigation into the role of NSUN6 in regulating the occurrence and development of OC. Our results showed that NSUN6 was downregulated in OC tissues and cell lines, and its expression was negatively correlated with tumor stage, metastasis, and patient survival. Its overexpression suppressed cell proliferation, migration, and invasion, primarily via inhibition of AKT phosphorylation. Combination treatment with the AKT inhibitor MK2206 further enhanced these effects. Mouse models confirmed a reduced tumor burden upon NSUN6 overexpression. Additionally, NSUN6 showed significant associations with cytokine signaling and immune cell infiltration.

## Results

### Integrative analysis identifies NSUN6 as a prognostic biomarker in ovarian cancer

**Fig. 1:**
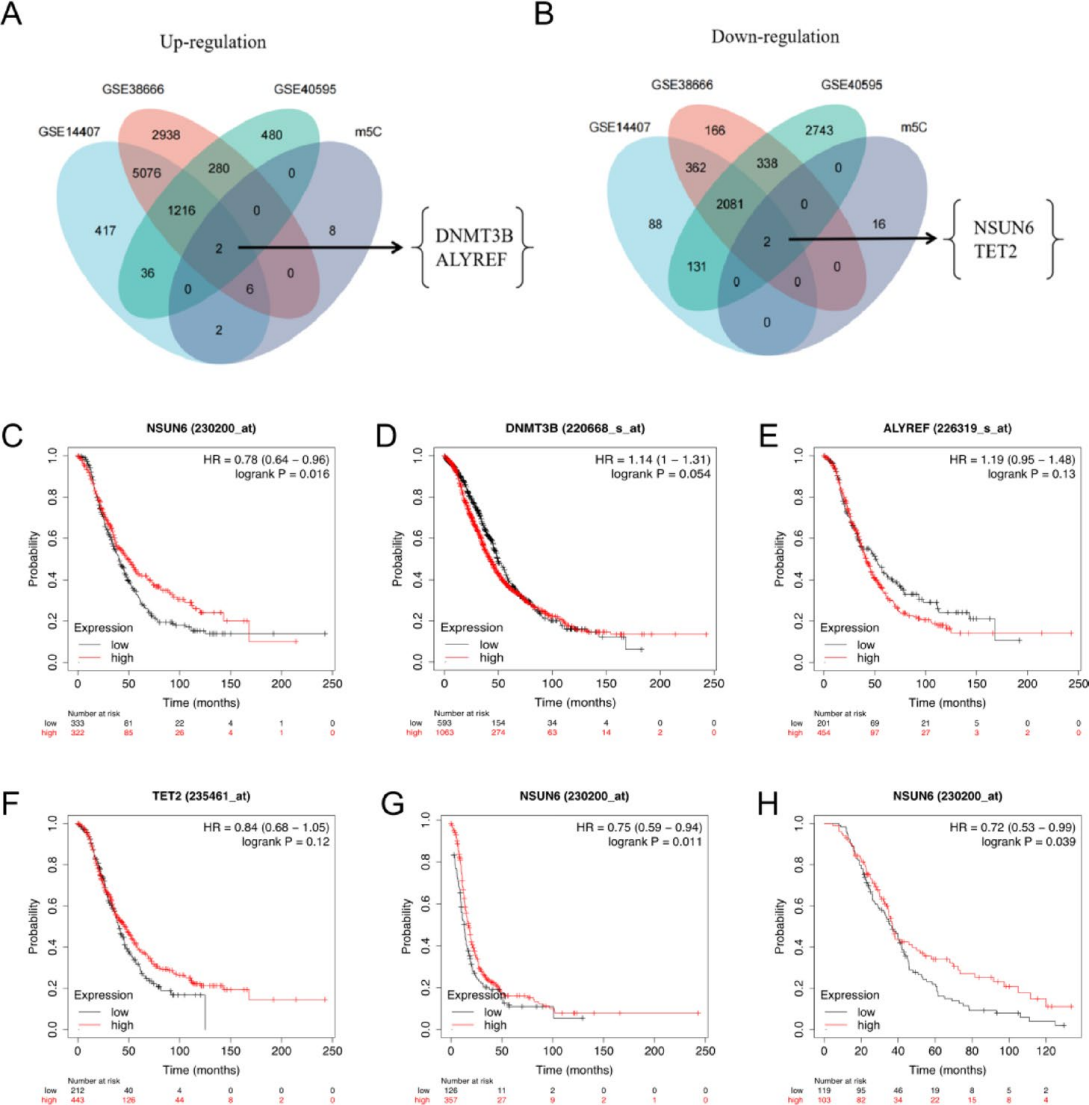
Integrative analysis identifies NSUN6 as a prognostic biomarker in ovarian cancer. (**A**) DNMT3B and ALYREF were identified as the up-regulated overlapping genes between m⁵C related genes and the differentially expressed genes (DEGs). (**B**) NSUN6 and TET2 were identified as the two down-regulated overlapping genes between m⁵C related genes and the DEGs. (**C**-**F**) Kaplan-Meier analysis of overall survival (OS) in patients with differential expression of each m⁵C related gene. (**G**) Kaplan-Meier analysis of post-progression survival (PPS) in patients with differential expression of NSUN6. (**H**) Kaplan-Meier analysis of progression-free survival (PFS) in patients with differential expression of NSUN6.

To identify potential oncogenic drivers in ovarian cancer, we performed comparative transcriptomic analysis of ovarian cancer versus normal ovarian tissues across three GEO datasets (GSE14407, GSE38666, GSE40595) using stringent criteria (*P* < 0.05, |log2FC|>1). This differential expression analysis revealed 417 upregulated and 88 downregulated genes in GSE14407, 2938 upregulated and 166 downregulated genes in GSE38666, and 480 upregulated versus 2743 downregulated genes in GSE40595 (Supplementary Table S1). These identified differentially expressed genes (DEGs) may functionally contribute to ovarian carcinogenesis through distinct oncogenic pathways. Given the established role of m⁵C methylation in cancer epigenetics, we integrated these DEGs with a curated m⁵C related genes dataset (Supplementary Table S2). Venn diagram analysis identified two significantly upregulated m⁵C regulators (DNMT3B, ALYREF) and two downregulated genes (NSUN6, TET2), all of which are critical m⁵C-related molecules involved in ovarian cancer development and progression (Fig. 1A-B).

The Kaplan-Meier Plotter was utilized to assess the prognostic value of mRNA expression levels of the four aforementioned genes in ovarian cancer (OC). We found that the low expression of NSUN6 was significantly associated with a shorter overall survival (OS) (Fig. 1C), whereas DNMT3B, ALYREF, and TET2 showed no significant correlation with prognosis (Fig. 1D-F). Additionally, patients with low NSUN6 expression exhibited worse post-progression survival (PPS) and progression-free survival (PFS) (Fig. 1G-H). These findings suggest that NSUN6 may serve as a promising prognostic biomarker in OC, warranting further functional studies to explore its biological and clinical implications in OC pathogenesis.

### Low NSUN6 expression in OC and its clinicopathological relevance

**Fig. 2:**
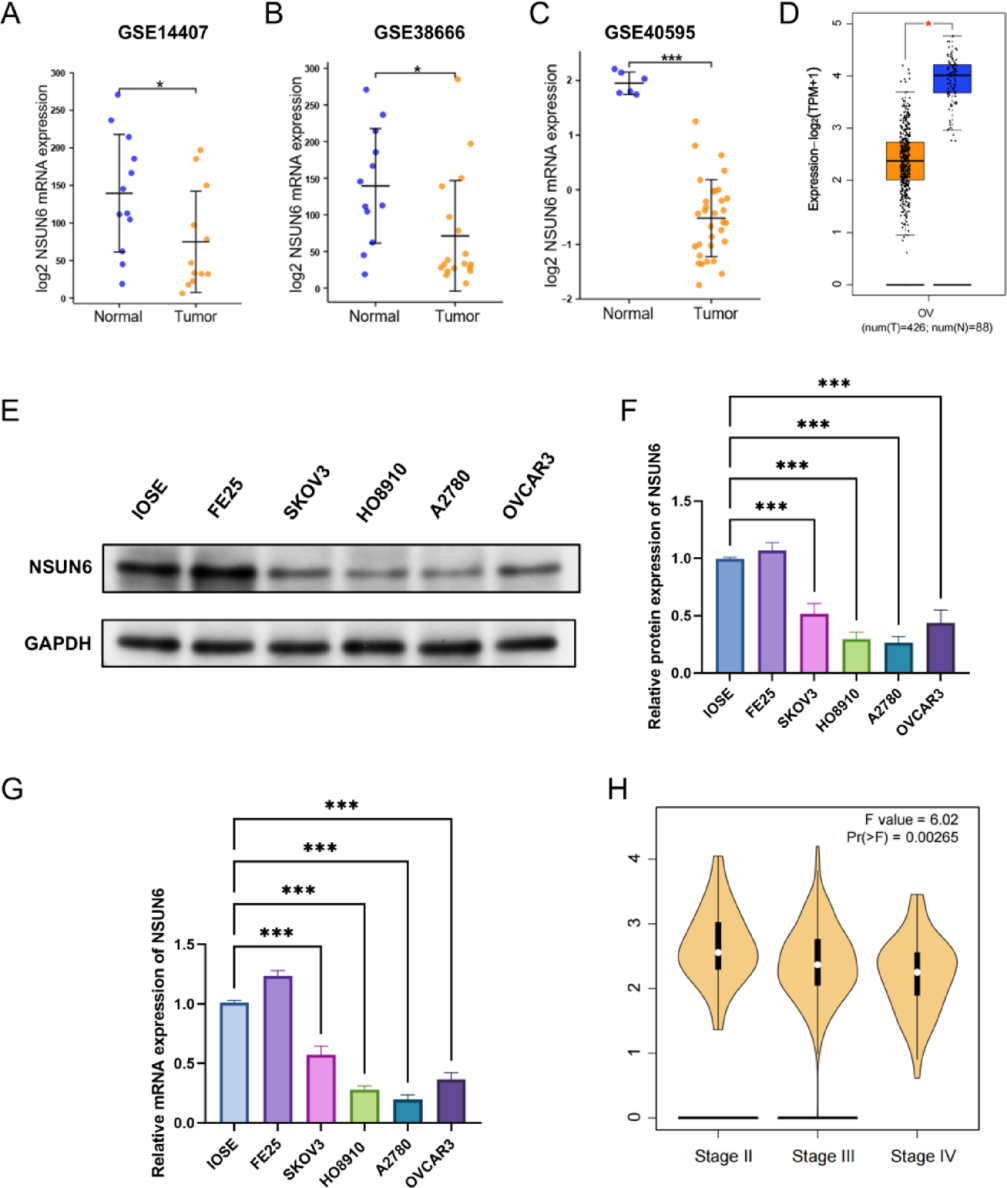
Low NSUN6 expression in OC and its clinicopathological relevance. (**A**-**C**) The mRNA expression of NSUN6 in GSE14407, GSE38666, and GSE40595. (**D**) The mRNA expression of NSUN6 in OC from GEPIA 2.0 database. (**E**, **F**) The protein expression levels of NSUN6 in OC cell lines. (**G**) The mRNA expression levels of NSUN6 in OC cell lines. (**H**) The correlation between NSUN6 expression and the stages of OC (GEPIA 2.0). *P < 0.05, **P < 0.01, ***P < 0.001.

Using several datasets and in vitro experiments to further investigate the expression level of NSUN6 in OC. First, we compared NSUN6 transcript levels among three OC GEO datasets and normal ovarian tissues. The results exhibited that compared with normal tissue, the mRNA level of NSUN6 in OC was significantly decreased (Fig. 2A-C). GEPIA2 analysis confirmed these findings (Fig. 2D) In addition, in vitro experiments were performed to validate the bioinformatics results. Western blot and quantitative PCR analyses demonstrated that both protein and transcription expression of NSUN6 were markedly downregulated in ovarian cancer tissues compared to normal tissues (Fig. 2E-G). Next, we explored the relationship between the NSUN6 expression and clinicopathological features of OC patients. The GEPIA2 analysis revealed that NSUN6 expression was correlated with the cancer stage of OC. Progressive downregulation of NSUN6 expression was observed with increasing tumor grade (Fig. 2H).

### Expression and clinical significance of NSUN6 in ovarian tissues

**Fig. 3:**
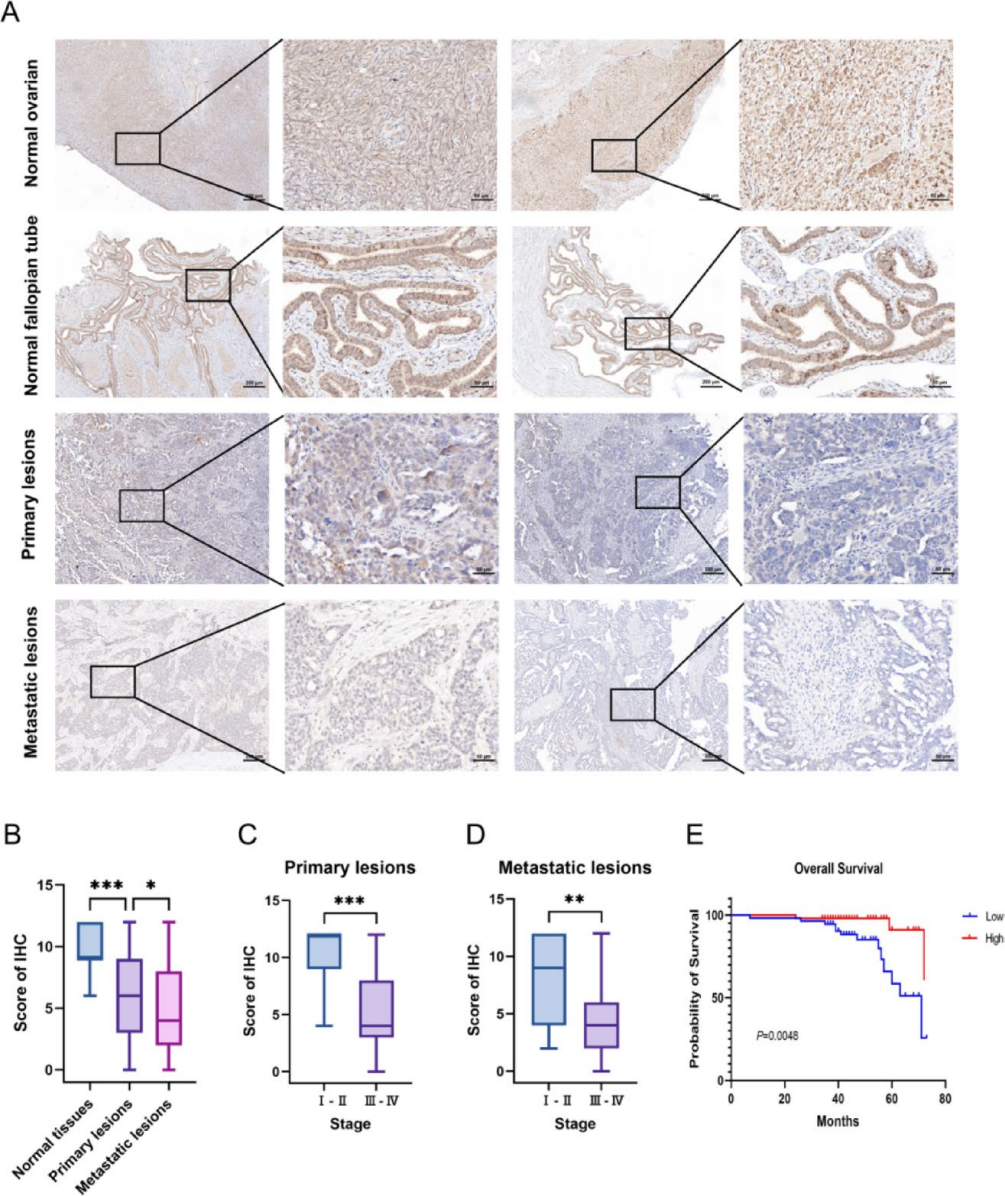
Expression and clinical significance of NSUN6 in ovarian tissues. (**A**) Immunohistochemistry (IHC) of NSUN6 in normal ovaries, normal fallopian tubes, primary and metastatic ovarian cancer lesions. (**B**) NSUN6 IHC scores of normal tissues, primary ovarian cancer lesions, and metastatic lesions. (**C**) NSUN6 IHC scorse of primary ovarian cancer lesions at different stages. (**D**) NSUN6 IHC scorse of metastatic ovarian cancer lesions at different stages. (**E**) The relationship between the protein levels of NSUN6 and overall survival in ovarian cancer patients. **P < 0.01, ***P < 0.001.

To assess the expression of NSUN6 in OC patients, we performed immunohistochemical (IHC) staining on paraffin-embedded clinical specimens and quantitatively analyzed the staining intensity. IHC results showed that NSUN6 protein was weakly positive or negative in primary and metastatic lesions of OC, whereas strong expression was observed in normal ovarian and fallopian tube tissues (Fig. 3A). Quantitative scoring indicated that NSUN6 levels were significantly lower in metastatic lesions compared to primary OC tissues, and both were markedly lower than those in normal tissues (Fig. 3B). Moreover, NSUN6 expression negatively correlated with tumor stage in both primary and metastatic samples (Fig. 3C-D). We further analyzed the association between NSUN6 expression and clinical characteristics, including age, tumor stage, lymphatic metastasis, and lymphatic/distant metastasis. Low NSUN6 expression was strongly correlated with advanced tumor stage, metastasis, and younger patient age, suggesting a potential role of NSUN6 in tumor aggressiveness (Table 1).

Furthermore, Kaplan-Meier survival analysis showed that OC patients with high NSUN6 expression exhibited significantly longer overall survival (OS) compared to those with low expression levels (Fig. 3E). Then, Cox regression analysis was performed to evaluate the association between various clinicopathological variables and patient prognosis. Univariate Cox regression analysis identified low NSUN6 expression, lymphatic metastasis, and distant metastasis as prognostic risk factors in OC patients. Multivariate analysis revealed that these three factors that mentioned above were not independent prognostic factors (Supplementary Table S3). Together, these findings suggest that NSUN6 is downregulated in OC and may serve as a potential prognostic biomarker associated with favorable outcomes.

**Table 1:** The correlation between NSUN6 expression and clinical pathological features.

Characteristics	Numbers of case (%)	NSUN6 expression		*P* value
Age(y) Low High				
≥ 55	43(40.6)	27	16	0.029
< 55	63(59.4)	26	37	
Clinical stage				
Ⅰ-Ⅱ	19(17.9)	1	18	< 0.001
Ⅲ-Ⅳ	87(82.1)	47	40	
Lymphatic metastasis				
Yes	50(47.2)	35	15	< 0.001
No	56(52.8)	20	36	
Distant metastasis				
Yes	25(23.6)	21	4	< 0.001
No	81(76.4)	34	47	

### NSUN6 inhibits the proliferation, migration and invasion of OC cells

**Fig. 4:**
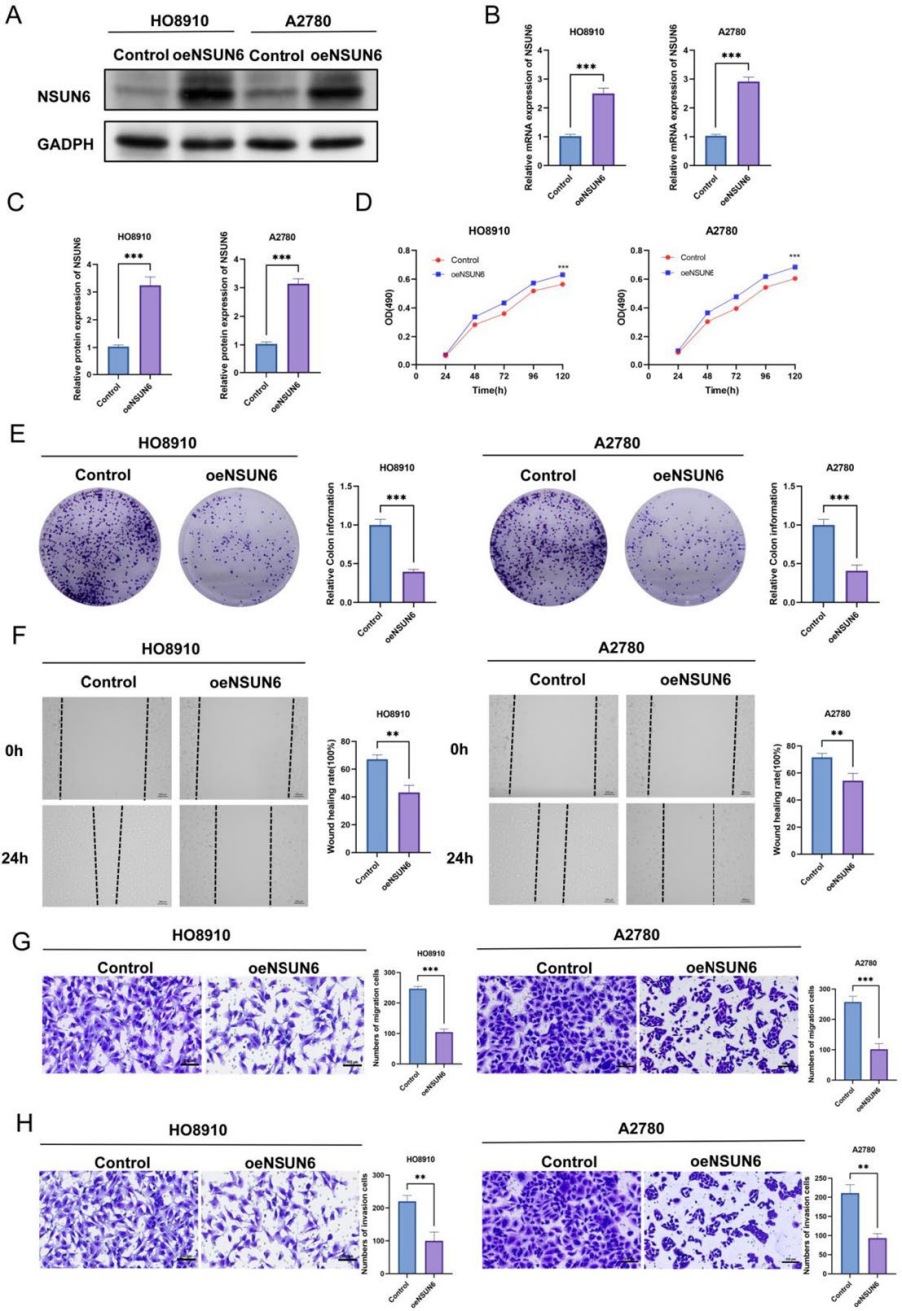
NSUN6 overexpression inhibits the proliferation, migration, and invasion of ovarian cancer cell lines. (**A**-**B**) NSUN6 protein expression following transfection with overexpression plasmids in HO8910 and A2780 cells. (**C**) NSUN6 mRNA expression following transfection with overexpression plasmids in HO8910 and A2780 cells. (**D**) CCK8 assay performed after NSUN6 overexpression in HO8910 and A2780 cells. (**E**) Colony formation assay performed after NSUN6 overexpression in HO8910 and A2780 cells. (**F**) Scratch wound-healing assay performed after NSUN6 overexpression in HO8910 and A2780 cells. (**G**) Transwell migration assay performed after NSUN6 overexpression in HO8910 and A2780 cells. (**H**) Transwell invasion assay performed after NSUN6 overexpression in HO8910 and A2780 cells. **P < 0.01, ***P < 0.001.

To elucidate the functional role of NSUN6 in ovarian cancer (OC) progression, we performed a series of in vitro assays to evaluate its impact on cellular proliferation, migration, and invasion. NSUN6 was overexpressed in OC cell lines, and successful upregulation was confirmed via qPCR and western blotting (Fig. 4A-C). CCK-8 assays revealed a significant reduction in cell proliferation upon NSUN6 overexpression (Fig. 4D). Colony formation assays further demonstrated a marked decrease in the number of colonies formed compared to controls (Fig. 4E). Additionally, scratch wound-healing assays showed impaired migration in NSUN6-overexpressing cells (Fig. 4F), a finding corroborated by transwell migration assays (Fig. 4G). Transwell invasion assays revealed a similar inhibitory effect on invasive potential (Fig. 4H). Collectively, these results suggest that NSUN6 functions as a suppressor of OC cell malignancy by inhibiting proliferation, migration, and invasion.

### Overexpression of NSUN6 inhibits OC progression by suppressing AKT activity

**Fig. 5:**
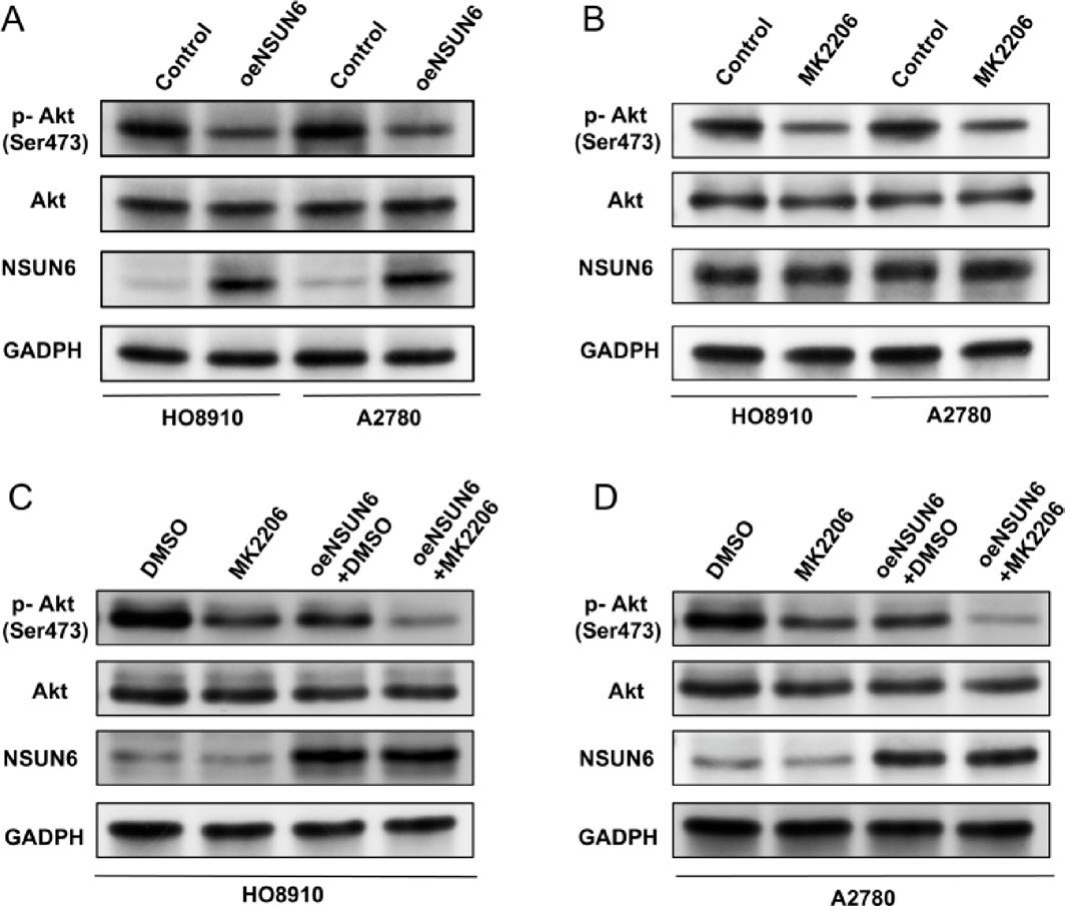
NSUN6 inhibits AKT activity. (**A**) The protein expression of NSUN6, p-AKT (S473) and AKT in two ovarian cancer cell lines after NSUN6 overexpression. (**B**) The protein expression of NSUN6, p-AKT and AKT in HO8910 and A2780 treated with DMSO and MK2206. (**C**,**D**) The protein expression of NSUN6, p-AKT and AKT in HO8910 and A2780 treated with DMSO, MK2206, NSUN6 plasmid, and NSUN6 plasmid plus MK2206.

Studies have shown that the PI3K/AKT signaling pathway is critically involved in OC progression. To determine whether NSUN6 exerts its tumor-suppressive effects through the PI3K/AKT signaling pathway, we assessed the phosphorylation status of AKT in NSUN6-overexpressing OC cells. Western blot revealed that overexpression of NSUN6 reduced the levels of phosphorylated AKT (p-AKT) in OC cells, while total AKT levels remained unchanged (Fig. 5A). In addition, treatment with MK2206, a selective AKT inhibitor, effectively reduced p-AKT levels without affecting NSUN6 expression, suggesting that NSUN6 may act upstream of AKT phosphorylation (Fig. 5B). Furthermore, combined treatment with NSUN6 overexpression and MK2206 resulted in a more pronounced decrease in p-AKT levels than either treatment alone (Fig. 5C and D).

**Fig. 6:**
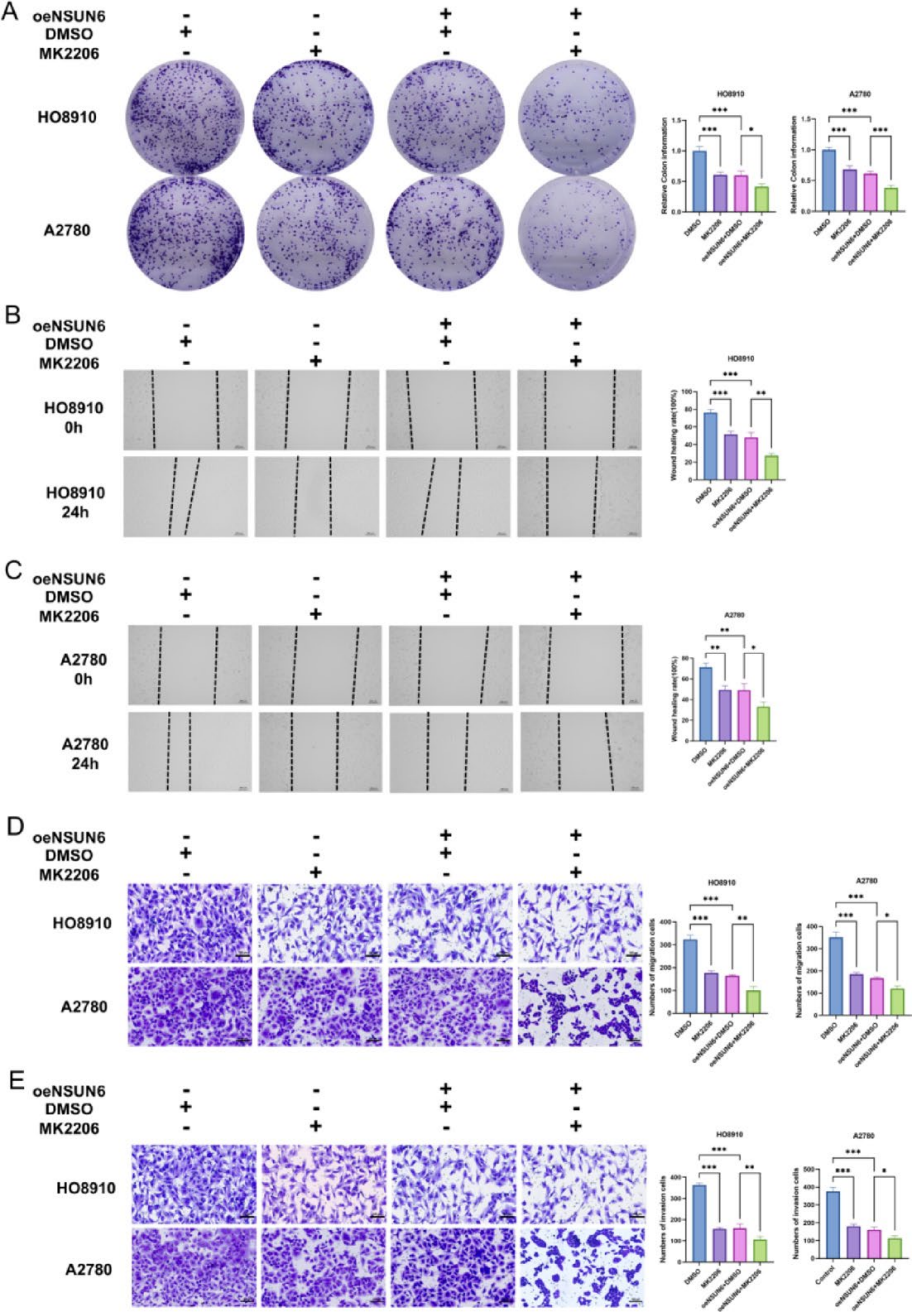
Overexpression of NSUN6 inhibits OC progression by suppressing AKT activity. (**A**) Colony formation assay in HO8910 and A2780 cells treated with DMSO, MK2206, NSUN6 plasmid, and NSUN6 plasmid plus MK2206. (**B**-**C**) Scratch wound-healing assay in HO8910 and A2780 cells treated with DMSO, MK2206, NSUN6 plasmid, and NSUN6 plasmid plus MK2206. (**D**) Transwell migration assay in HO8910 and A2780 cells treated with DMSO, MK2206, NSUN6 plasmid, and NSUN6 plasmid plus MK2206. (**E**) Transwell invasion assay in HO8910 and A2780 cells treated with DMSO, MK2206, NSUN6 plasmid, and NSUN6 plasmid plus MK2206. *P < 0.05, **P < 0.01, ***P < 0.001.

We next investigated how NSUN6-mediated regulation of AKT affects ovarian cancer cell behavior. Colony formation assays showed that both NSUN6 overexpression and MK2206 inhibited colony formation, with the strongest suppression observed under combinatorial treatment (Fig. 6A). Consistent findings were observed in scratch wound-healing and transwell migration assays, where co-treatment led to the greatest inhibition of cell migration (Fig. 6B-D). Similarly, transwell invasion assays demonstrated that both NSUN6 and MK2206 independently suppressed invasion, with combination treatment producing the most significant inhibitory effects (Fig. 6E). Collectively, these results suggest that NSUN6 inhibits the metastatic behavior of OC cells at least in part by downregulating AKT phosphorylation.

### In vivo experiments show that NSUN6 inhibits OC progression

**Fig. 7:**
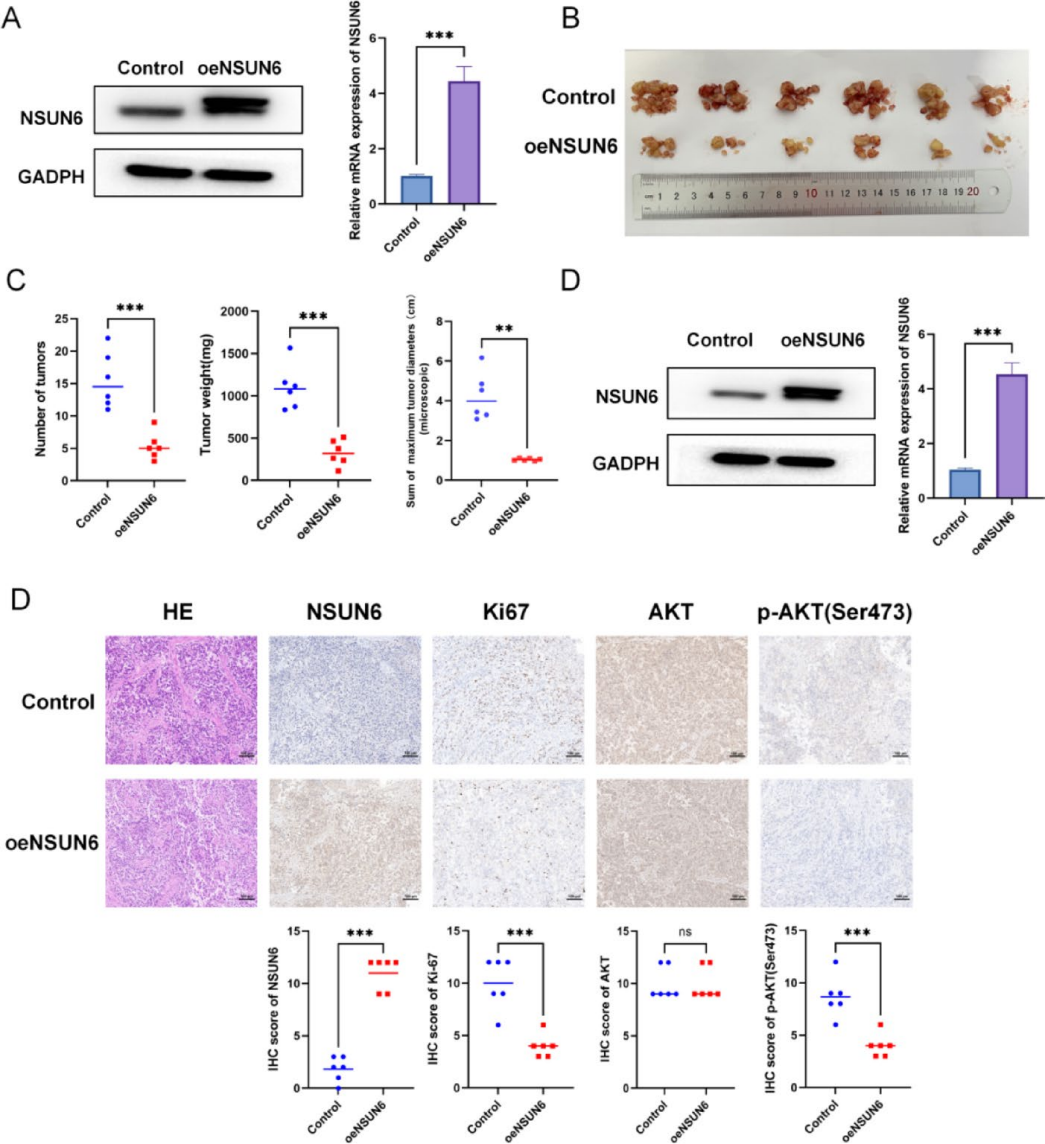
The effects of NSUN6 overexpression on intraperitoneal tumor growth in OC in vivo. (**A**) Protein and mRNA expression levels of NSUN6 after overexpression and control lentivirus transfection in HO8910 cells. (**B**) Tumors harvested from mice on day 28 after intraperitoneal injection. (**C**) Statistical analysis of tumor number, tumor weight, and the sum of maximum tumor diameters in mice. (**D**) Protein and mRNA expression level of NSUN6 in tumor tissues. (**E**) Immunohistochemical analysis of NSUN6、ki67、AKT、p-AKT (Ser473) expression in control and NSUN6-overexpressing mouse tissues. **P < 0.01, ***P < 0.001.

To investigate the biological function of NSUN6 in vivo, HO8910 ovarian cancer cells stably overexpressing NSUN6 or empty vector were established (Fig. 7A). Subsequently, these cells were injected intraperitoneally or subcutaneously into nude mice to evaluate peritoneal dissemination and subcutaneous tumor growth, respectively. For the intraperitoneal model, after four weeks, the mice were sacrificed and tumor nodules were harvested (Fig. 7B). Quantitative analysis revealed that the number of tumor nodules, tumor weight, and the sum of maximum tumor diameters were significantly reduced in the NSUN6-overexpression group compared to the control group (Fig. 7C). Western blot and qRT-PCR analyses confirmed sustained overexpression of NSUN6 in tumor tissues (Fig. 7D). Similarly, in the subcutaneous model, NSUN6 overexpression significantly suppressed tumor growth, as evidenced by reduced tumor volume and weight (Supplementary Figure S1A, B). Furthermore, immunohistochemical staining consistently showed weaker Ki-67 signals and reduced p-AKT (Ser473) expression in tumors derived from the NSUN6-overexpressing group in both intraperitoneal and subcutaneous models, while total AKT levels remained unchanged (Fig. 7E, Supplementary Figure S1C). Collectively, these findings demonstrate that NSUN6 suppresses both peritoneal and subcutaneous tumor growth in vivo, further supporting its tumor-suppressive role in ovarian cancer progression.

### Functional and immunological role of NSUN6 in ovarian cancer

To elucidate the functional significance of NSUN6 in ovarian cancer (OC), we conducted Gene Ontology (GO) and KEGG pathway enrichment analyses. GO annotation revealed that NSUN6 was primarily linked to biological processes such as cytokine-mediated signaling, leukocyte migration, and cellular divalent inorganic cation homeostasis (Supplementary Figure S1A). The top enriched cellular components included collagen-containing extracellular matrix, vesicle lumen, and cytoplasmic vesicle lumen (Supplementary Figure S1B). For molecular functions, NSUN6 was predominantly involved in signaling receptor activator activity, receptor ligand activity, and G protein-coupled receptor binding (Supplementary Figure S1C). KEGG pathway analysis further identified neuroactive ligand-receptor interaction, cytokine-cytokine receptor interaction, and chemokine signaling pathway as the most significantly enriched pathways (Supplementary Figure S1D).

Given the critical role of immune infiltration in tumor progression, we further assessed the relationship between NSUN6 expression and immune cell populations using the Xiantao platform. NSUN6 expression was significantly correlated with several immune infiltrates in OC (Supplementary Figure S1E). Specifically, it was positively associated with T central memory (TCM) and natural killer (NK) cells, while negatively correlated with cytotoxic T cells and T helper 1 (Th1) cells (Supplementary Figure S1F). These findings were further validated in our cohort of ovarian cancer tissue specimens. Our results showed that NSUN6 expression was significantly negatively correlated with CD4^+^ T cells (*r* = −0.2166, *P* = 0.0258) and CD8^+^ T cells (*r* = −0.2286, *P* = 0.0184), and significantly positively correlated with CD56^+^ NK cells (*r* = 0.2398, *P* = 0.0133) (Supplementary Figure S3A, B). These findings suggest that NSUN6 may influence OC progression through both immune regulation and signaling pathways.

## Discussion

Ovarian cancer (OC) is one of the most prevalent and lethal malignancies of the female reproductive system^33^, characterized by high incidence and mortality rates worldwide^34^. In recent years, advances in treatment modalities—including anti-angiogenic therapy and PARP inhibitors—have contributed to modest improvements in patient prognosis beyond conventional surgery and chemotherapy^35,36^. Nonetheless, the majority of ovarian cancer cases are diagnosed at an advanced stage (FIGO stage III or IV), resulting in persistently poor clinical outcomes, with a five-year survival rate remaining dismally low^37,38^. These challenges underscore the urgent need to identify novel molecular biomarkers and therapeutic targets to enhance early diagnosis and improve clinical management of ovarian cancer.

RNA methylation, a widespread post-transcriptional modification, occurs in both eukaryotic and prokaryotic organisms^39^. Recently, emerging evidence has demonstrated its involvement in key biological processes, including tumor immunity^40,41^, cancer progression^42,43^ and other pathophysiological processes. Among various methylation types, 5-methylcytosine (m⁵C) is a well-characterized RNA modification associated with the progression of several malignancies, such as lung cancer^44^, gastric cancer^23^, hepatocellular carcinoma^45^ and other malignancies. NSUN6, a member of the RNA: m⁵C methyltransferase (MTase) family, has been identified as a methyltransferase present in the cytoplasm that methylates C72 of tRNAThr and tRNACys^46^ and plays an important role in the development and prognosis of various cancers^47,48^.

In this study, we first screened three publicly available ovarian cancer (OC) datasets along with a curated list of m⁵C-related genes, identifying four co-DEGs: DNMT3B and ALYREF expression were upregulated, while TET2 and NSUN6 were downregulated in OC compared with normal ovarian tissues. Using the Kaplan-Meier Plotter, we analyzed the survival correlation of these co-DEGs in OC patients. Among the four genes, only low NSUN6 expression was significantly associated with reduced overall survival (OS), whereas DNMT3B, ALYREF, and TET2 showed no significant correlation. Further survival analysis revealed that NSUN6 expression was significantly associated with post-progression survival (PPS), but not progression-free survival (PFS), suggesting its potential as a prognostic biomarker.

Although no prior studies have explored NSUN6 in OC, previous reports have demonstrated its critical role in other cancers. For instance, there has been evidence that NSUN6 overexpression can inhibit the growth and invasion of pancreatic cancer cells and evaluate the prognosis of pancreatic cancer patients^29^. Moreover, NSUN6 can also be used as a prognostic marker in cutaneous melanoma and colorectal cancers and is associated with tumour immune infiltration^49,50^. Given its distinct survival association compared to the other three co-DEGs, we selected NSUN6 for further functional validation.

We collected paraffin-embedded ovarian cancer tissue specimens from patients and analyzed NSUN6 expression levels, as well as their association with clinicopathological characteristics and patient prognosis. IHC analysis revealed that NSUN6 expression was significantly reduced in OC tissues, particularly in metastatic lesions, and negatively correlated with tumor stage and clinical aggressiveness. Low NSUN6 expression was associated with shorter overall survival and served as a prognostic risk factor in univariate Cox analysis. However, NSUN6 was not a significant prognostic factor in multivariate analysis after adjusting for tumor stage, lymph node metastasis, and distant metastasis. This indicates that its prognostic impact is confounded by its close association with advanced tumor stage and metastatic status, which are the dominant prognostic determinants in ovarian cancer. Other potential confounding factors may include residual tumor volume and histological grade. Therefore, NSUN6 should not be considered an independent prognostic biomarker in clinical practice without further validation. Future prospective studies with larger, multi-center cohorts, stage‑stratified analyses, and detailed treatment information are needed to determine whether NSUN6 may have prognostic utility in specific clinical contexts, such as in early-stage patients or within defined molecular subtypes.

Furthermore, in vitro experiments confirmed that NSUN6 is significantly downregulated in ovarian cancer (OC) tissues and functions to suppress OC cell proliferation, invasion, and migration. These findings provide the first experimental evidence supporting the tumor-suppressive role of NSUN6 in OC. The PI3K/AKT/mTOR pathway, frequently dysregulated in cancer^51,53,53^, is a key mediator of tumor progression. A previous study reported that NSUN6 deficiency promoted osteosarcoma progression by downregulating EEF1A2 and activating the AKT/mTOR signaling cascade^30^. In line with this, our data showed that NSUN6 overexpression reduced AKT phosphorylation and inhibited malignant cellular behaviors in OC. These inhibitory effects were further validated through in vivo experiments, reinforcing NSUN6’s role as a potential therapeutic target in OC. However, the precise molecular mechanism by which NSUN6 regulates AKT activity remains to be fully elucidated. Given that NSUN6 is an RNA m⁵C methyltransferase primarily acting on tRNAs and specific mRNAs, we speculate that it may indirectly regulate AKT signaling by affecting the stability or translation efficiency of key genes in the PI3K/AKT pathway. That said, whether NSUN6 exerts its tumor-suppressive effects through its m⁵C methyltransferase activity, specific RNA targets, protein-protein interactions, or other non-enzymatic pathways remains to be determined. Moreover, the downstream effector molecules through which NSUN6-inhibited p-AKT suppresses OC cell proliferation, migration, and invasion have not yet been characterized. The primary focus of this study is to reveal the phenotypic functions and prognostic value of NSUN6 in ovarian cancer, and we fully recognize that these mechanistic aspects have not been explored in depth here. Therefore, we will conduct future studies using methods such as m⁵C sequencing, RNA stability assays, catalytic domain mutations, and co-immunoprecipitation to identify the potential RNA targets and interacting partners of NSUN6, as well as to determine whether its enzymatic activity is required for its tumor-suppressive function. In addition, downstream effectors of the AKT pathway should be examined to determine whether they are modulated by NSUN6 in an AKT-dependent manner. These efforts will help clarify whether m⁵C-dependent or -independent mechanisms contribute to its role in OC progression and provide a more comprehensive understanding of its tumor-suppressive function.

Moreover, loss-of-function assays were not successfully performed in this study. Despite multiple attempts using different siRNA sequences, efficient NSUN6 knockdown could not be achieved, likely due to its already low baseline expression in OC cells. Therefore, the current study focuses on overexpression phenotypes and clinical correlations. Future studies employing alternative strategies (e.g., CRISPR or stable knockdown in higher-expression cell lines) are needed to confirm NSUN6 endogenous tumor-suppressive role.

Additionally, KEGG pathway enrichment analysis revealed that NSUN6 is implicated in the chemokine signaling pathway. Chemokines are small secreted proteins that bind to G protein-coupled receptors (GPCRs), regulating diverse cellular processes and playing a pivotal role in tumorigenesis and malignant progression^54,55^. Therefore, it is reasonable to believe that NSUN6 may partially inhibit the malignant biological behaviour of OC through chemokine signaling pathway. This finding suggests that NSUN6 is closely associated with the proliferation and invasion of OC cells and plays a significant role in the OC malignant progression.

More and more evidences demonstrate that the malignant phenotype of cancer including OC, is linked to components within the tumor microenvironment (TME)^56,57^, particularly tumor-infiltrating immune cells^58,59^. Comprehensive analysis of tumor-infiltrating immune cells (TIICs) can elucidate mechanisms of immune evasion, identify novel therapeutic targets, and inform innovative treatment strategies^60,61^. NK cells play a critical role in tumor immunosurveillance, especially in eliminating early tumor and metastasis^62^. Similarly, TCM cells also have better anti-tumor immunity than TEM cells^63^. In this study, we assessed the correlation between NSUN6 expression and TIICs in OC using public databases, and found that NSUN6 was positively correlated with infiltrating TCM cells and NK cells, and a negative correlation with cytotoxic cells and TH1 cells. These findings were further validated in our cohort of ovarian cancer tissue specimens. However, the specific molecular mechanisms by which NSUN6 regulates immune cell infiltration remain largely unclear. We hypothesize that NSUN6 may modulate the tumor immune microenvironment by affecting chemokine secretion, regulating immune cell proliferation, apoptosis, or other biological processes. Given the complexity of the tumor immune microenvironment, further studies, including chemotaxis assays, co-culture systems, and in-depth profiling of immune cell subsets in vivo, are warranted to dissect the precise immunoregulatory functions of NSUN6. These investigations will help clarify whether NSUN6 can serve as a potential therapeutic target for combination immunotherapy in ovarian cancer.

In summary, our study demonstrates that NSUN6 suppresses ovarian cancer (OC) progression by inhibiting AKT signaling, as supported by bioinformatics analysis, in vitro and in vivo experiments, and immunohistochemical validation. These findings provide novel insights into the functional role of NSUN6 in OC pathogenesis and establish a theoretical basis for its potential application in clinical diagnosis and targeted therapy.

## Materials and methods

### Data acquirement and analysis by bioinformatics tools

We obtained three ovarian cancer datasets GSE14407, GSE38666 and GSE40595 from Gene Expression Omnibus (GEO) database and downloaded the m5c related gene datasets. Then, we screened the overlapping differentially expressed genes in these four data sets through Venn Diagram.

The Kaplan-Meier plotter(http://kmplot.com/analysis/), an online public resource containing cancer genes as well as survival condition in different cancers, was used to analyze the prognosis of the four co-differentially expressed genes(co-DEGs) mentioned above in OC. Overall survival rate, progression free survival and post progression survival as the main indicators of prognosis in OC. We applied GEPIA2(http://gepia.cancer-pku.cn/) and UALCAN database (http://ualcan.path.uab.edu/index.html) to compare the expression of NSUN6 mRNA and protein in ovarian cancer and normal tissues and analyzed the differences in the expression of NSUN6 in each stage of OC.

Kyoto Encyclopedia of Genes and Genomes (KEGG) and Gene Ontology (GO) including biological process, cell composition and molecular function were used to enrich and analyze the biological function and pathway of NSUN6 in patients with OC^64^. Furthermore, we analyzed the correlation between NSUN6 expression in OC and immune infiltrating cells using XianTao Tools (https://www.xiantaozi.com/), a comprehensive online bioinformatic analysis software, in conjunction with the TCGA-OC database.

### Cell culture

All cell lines used in this study include human ovarian cancer cells OVCAR3 HO8910, SKOV3, A2780, and normal ovarian cells IOSE and FE25 are all from the School of Basic Medicine, Central South University. All cell lines were cultured in RPMI-1640 medium containing 10% fetal bovine serum and incubated at 37 °C in a 5% CO2 humidified atmosphere.

### Construction of NSUN6 overexpressing cell lines

NSUN6 overexpression plasmid pcDNA3.1-NSUN6-3Flag-Puro purchased from HonorGene (HG-HO182543). Under the participation of lipofectamine 2000, the NSUN6 overexpression plasmid was transfected into A2780 and HO8910 cell lines for 24 h. Perform Western blot and qPCR to validate the efficacy of transfection.

Lentiviruses with 3FIAG tag and puromycin resistance were purchased from Shanghai Genechem Co., Ltd. The MOI for infecting HO8910 is 10. Stably transfect cells with NSUN6 overexpressing lentivirus and corresponding negative control. After 72 h, screen stable transfected HO8910 with puromycin (2 µg/ml) for subsequent experiments.

### qRT-PCR and Western blot

Total RNA was extracted from cells and tumors using the TRIzol Reagent. Reverse transcription was performed using PrimeScript RT Master Mix (YEASEN). Perform qRT-PCR using SYBR green mixture (Toyo Textile) and CFX96TM real-time PCR detection system (Bio Rad). The NSUN6 primer was: F 5’-GAAAGGCATGGGCATAAGAA-3’, R 5’-TGTGTGTTGTTTTCCCTCCA-3’. The GADPH primer was: F 5’-TGAAGGTCGGAGTCAACGGATTTGGT-3’, R 5’-CATGTGGGCCATGAGGTCCACCAC-3’.

The total protein was extracted from cells with RIPM buffer, quantified with BCA kit (Thermo), and then separated on 10% gel by SDS-PAGE. Then imprint it on the PVDF membrane. After sealing with 5% skim milk, the membrane was incubated overnight with the primary antibody at 4 °C, and then incubated with the HRP conjugated secondary antibody at room temperature for 2 h. Then, use a chemiluminescence imaging system to detect immune reactions. The following antibodies were used: NSUN6 (Ab307430, Abcam), GADPH (60004, Proteintech), p-AKT (T40067, Abmart), AKT (4691, CST).

### Cell Counting Kit-8 (CCK-8) and colony formation assay

Perform CCK-8 and colony formation assays to measure the proliferation of stably transfected OC cells. For CCK-8 assay, cells were seeded at a density of 2000 cells/well in a 96 well plate, and the optical density values of each well were detected at 450 nm using an enzyme-linked immunosorbent assay reader at 24, 48, 72 and 96 h after inoculation.

For colony formation assay, cells were seeded at a density of 1000 cells/well in a 6-well plate and cultured for 2 weeks. Then, fix the cells in 10% paraformaldehyde for 10 min and stain with 1% crystal violet for 30 min. Subsequently, take photos and count the colonies. A colony is defined as greater than 50 cells. Quantify colonies using ImageJ V2.6.

### Transwell assay and wound healing assay

For the transwell invasion assay, the upper membrane was pre-coated with 100 µl of matrix gel. Cells were resuspended in FBS-free medium and inoculated in the upper chamber (1 × 10^5^/well), and medium containing 20% FBS was added to the lower chamber. 24 h later, cells were fixed with 4% paraformaldehyde and stained with 0.1% crystal violet. Stained cells were counted in three randomly selected areas using a light microscope. Transwell migration experiments were as described above except that no matrix gel was added.

For wound healing assay, cells were seeded into a 6-well plate. When the cells are completely confluent, scratch the cell layer with a 200 µl sterile pipette tip and wash with FBS. Maintain the cells in a FBS-free culture medium and take images to record the wound width at 0 and 24 h.

### Patients and specimens

106 primary lesion samples and 78 metastatic lesion samples from ovarian cancer patients, 9 normal ovarian samples, and 9 normal fallopian tube samples obtained from Xiangya Hospital of Central South University from 2016 to 2022. Collect specimens according to the ethical standards of Xiangya Hospital, Central South University. The Ethics Committee of Xiangya Hospital of Central South University has approved this study. This study was approved by the Ethics Committee of Xiangya Hospital, Central South University(2025081825), in accordance with the Declaration of Helsinki. All pathological specimens were retrospectively obtained from medical records or biological samples collected during prior clinical diagnosis and treatment. Subject identifiers were de-identified and will remain anonymous in all research outputs. The Ethics Committee granted a waiver of informed consent due to the use of de-identified archival specimens.

### Immunohistochemistry

After dewaxing the tissue sections in turpentine and hydrating them in a gradient of alcohols (95%, 85%, 70%), the exposure of tissue antigens was restored using citrate buffer for 3 min. Endogenous peroxidase activity was blocked using 3% H_2_O_2_ for 20 min. The sections were incubated with NSUN6 antibody (Ab307430, Abcam) at 37 °C for 1 h and then with a secondary antibody for 30 min. Subsequently, the chromogenic reaction was analyzed using DAB solution.

### Tumor xenograft model

A total of 24 4-week-old female BALB/c nude mice were purchased from the Animal Experiment Center of Central South University. The mice were randomly divided into two groups and injected with HO8910 cells stably transfected with either NSUN6-overexpressing lentivirus or negative control lentivirus. For the intraperitoneal model, cells were injected intraperitoneally, and after four weeks, the mice were anesthetized with CO₂and then euthanized via cervical dislocation. Tumor nodules were harvested for RNA and protein extraction. For the subcutaneous model, cells were injected subcutaneously into the flank of the mice. Four weeks later, the mice were euthanized, and subcutaneous tumors were excised for volume and weight measurement. All animals procedures were approved by the the Ethics Committee of the Experimental Animal Center of Central South University(CSU-2024-0390), following ARRIVE guidelines. IACUC of Animal research approved by the Ethics Committee of the Experimental Animal Center of Central South University (CSU-2024-0390).

### Statistical analysis

Compare two groups and multiple groups using student t-test and one-way ANOVA, respectively. Perform multiple comparisons at appropriate times. A p-value < 0.05 is considered statistically significant, and all tests are double tailed. All statistical tests were conducted using GraphPad Prism 8.0.

## Supplementary Information

Below is the link to the electronic supplementary material.

Supplementary Material 1

Supplementary Material 2

## Data Availability

The datasets used and/or analysed during the current study available from the corresponding author on reasonable request.
